# High Neuroticism Is Related to More Overall Functional Problems and Lower Function Scores in Men Who Had Surgery for Non-Relapsing Prostate Cancer

**DOI:** 10.3390/curroncol29080459

**Published:** 2022-08-17

**Authors:** Alv A. Dahl, Sophie D. Fosså

**Affiliations:** 1 Department of Oncology, National Resource Center for Late Effects after Cancer Treatment, Oslo University Hospital, N-0424 Oslo, Norway; 2 Faculty of Medicine, University of Oslo, N-0318 Oslo, Norway

**Keywords:** prostate cancer, neuroticism, radical prostatectomy, overall problems, prospective study, generalized estimation equations

## Abstract

The personality trait of neuroticism is associated with adverse health outcomes after cancer treatment, but few studies concern men treated for prostate cancer. We examined men with high and low neuroticism treated with radical prostatectomy for curable prostate cancer without relapse. We compared overall problems and domain summary scores (DSSs) between these groups, and if high neuroticism at pre-treatment was a significant predictor of overall problems and DSSs at follow-up. A sample of 462 relapse-free Norwegian men self-rated neuroticism, overall problems, and DSSs by the EPIC-26 before surgery and at three years’ follow-up. Twenty-one percent of the sample had high neuroticism. Patients with high neuroticism reported significantly more overall problems and DSSs at pre-treatment. At follow-up, only overall bowel problems and urinary irritation/obstruction and bowel DSSs were different. High neuroticism was a significant predictor of overall bowel problems and bowel and irritation/obstruction DSSs at follow-up. High neuroticism at pre-treatment was significantly associated with a higher rate of overall problems both at pre-treatment and follow-up and had some significant predictions concerning bowel problems and urinary obstruction at follow-up. Screening for neuroticism at pre-treatment could identify patients in need of more counseling concerning later adverse health outcomes.

## 1. Introduction

After cancer therapy, patient-reported outcome measures (PROMs) of dysfunctions and associated problems (bother), often combined as adverse health outcomes (AHOs), have become increasingly popular as a supplement to doctors’ evaluations. Concerning PROMs for men with prostate cancer (PCa), two international working groups have recommended the use of the Expanded Prostate Cancer Index Composite 26-question Short Form (EPIC-26) [1,2].

PROMs cover the personal subjective health experiences of the patients. These experiences are influenced by their daily health-related activities and their current mental functioning. Personality represents a major element of such functioning, regularly defined as “enduring patterns of perceiving, relating to, and thinking about the environment and oneself” [3]. Personality traits are prominent aspects of personality, and after being established in adolescence, they remain relatively stable over time and various life situations as characteristic patterns of coping and interpersonal functioning [3]. Modern personality theory defines five basic personality traits, which are established during childhood and adolescence and remain stable during the rest of life. However, recent research implies that basic traits may be modified by interventions or traumas like cancer [4,5]. Neuroticism is the most important basic trait concerning health and disease and is defined as follows: “Neuroticism is the propensity to experience negative emotions, including anxiety, fear sadness, anger, guilt, disgust, irritability, loneliness, worry, self-consciousness, dissatisfaction, hostility, embarrassment, reduced self-confidence, and feelings of vulnerability, in reaction to various types of stress” [6]. Neuroticism has a skewed distribution in the general population, with 11 to 20% of males having high neuroticism depending on the cut-off level applied [7].

In the general population, a high neuroticism score predisposes to many somatic diseases, mental disorders, and premature death [8,9,10]. High neuroticism is also a predictor of emotional distress in cancer patients a year after diagnosis, but none of these studies concerned PCa patients [11].

Despite its obvious relevance for PCa patients, the relation between neuroticism and AHOs after surgery is covered by few studies. One prospective study by our research group showed that some neuroticism versus no neuroticism at pre-treatment predicted increased sexual bother (problems) one year after radical prostatectomy (RP) [12]. Another of our prospective studies concerned men treated with robot-assisted prostatectomy (RALP) or radiotherapy. At follow-up 24 months post-treatment, multivariate analyses showed that higher neuroticism at pre-treatment was significantly associated with urinary bother, a trend for significance with sexual bother, and no significant association with bowel bother [13].

No prospective studies known to us of PCa patients treated with RP have examined the impact of pre-treatment high neuroticism on patient-rated overall problems or domain summary scores (DSSs) at pre-treatment and at several years’ follow-up. To fill this gap available pre-treatment and three years’ post-treatment EPIC-26 overall problem ratings and DSSs were analyzed related to high and low levels of neuroticism at pre-treatment. We asked the following research questions: (1) Do men with high versus low neuroticism report more overall problems or significantly lower (worse) DSSs at both time points? (2) Is high neuroticism at pre-treatment a significant predictor of overall problems and DSSs three years after RP?

## 2. Material and Methods

### 2.1. Design

This multicenter study had a longitudinal design and attempted to evaluate AHOs prospectively after RP and radiotherapy. Between November 2008 and December 2009, PCa patients with planned RP were included in the Norwegian Urinary Cancer Group (NUCG) VII study for PCa [12]. Men aged ≤80 years at diagnosis and with no adjuvant treatment were eligible. We had no data on the type of RP performed in the individual patients, but most of them had been operated with an open approach without attempts at nerve sparing.

Neuroticism at pre-treatment and the AHOs at several time points were based on responses to a questionnaire. The questionnaire was completed before RP (pre-treatment), and patients completed mailed questionnaires one and three years after RP. In order to study the long-term effects, we only present findings from the pre-treatment and from the three years’ follow-up evaluations.

### 2.2. Scales

*The EPIC-26* is a PROM for rating PCa-related AHOs of the *last four weeks* covering urinary, bowel, sexual, and hormonal functional domains with four to six items each [14,15], but the last domain was omitted due to lack of hormone treatment of the sample. The EPIC-26 covers different degrees of dysfunction but also contains three items by which the patients rate their overall urinary, bowel, and sexual problems (Q5, Q7, and Q12). The overall problems and 20 other EPIC-26 items (Q1-Q13e) measuring dysfunctions had five scoring alternatives that are from zero to 100 points: “None” (100), “Very Small” (75), “Small” (50), “Moderate” (25), and “Big” (0). These ratings were dichotomized as “Hardly any problem” (scores 100 and 75, defined as reference) and “Problem present” (scores 50 to 0). Three EPIC-26 items (Q2, Q3, and Q9) had four scoring alternatives: “No dysfunction/no pad use” (100), “Occasional dysfunction/one pad per day” (67), “Frequent dysfunction/two pads per day” (33), and “Always dysfunction/three or more pads” (0), which were dichotomized as “Hardly any problem/dysfunction” (scores 100 and 67, reference) and “Problem” (scores 33 and 0).

We also calculated the DSSs of the urinary incontinence and irritation/obstruction, bowel, and sexual domains at pre-treatment and follow-up. Lower DSSs imply more dysfunction.

*Neuroticism* was measured by the patients’ responses to an abbreviated version of the Eysenck Personality Questionnaire (EPQ-18) using six items with responses of “yes” (1) and “no” (0) [16]. (See Supplementary Materials). Based on the right-skewed distribution of the sum score, we defined “high neuroticism” by scores 2–6 and “low neuroticism” by scores 0–1. This resulted in a group of 97 patients (21%) with high neuroticism and 365 (79%) with low neuroticism (reference). Cronbach’s coefficient alpha for neuroticism was 0.72.

### 2.3. Other Variables

The patients rated their *level of education* [≤12 years (short) versus >12 years (long, reference)], *relationship status* (paired (reference) versus non-paired), *work status* (paid work versus pensioned), and *co-morbidity* classified as zero (reference), one, or two or more coexisting somatic disease(s) among those listed in the EPIC-50 [17]. Based on data from the patients’ medical records, three *risk groups* were defined, and low risk was referenced [18]. *Nerve sparing* of unilateral or bilateral neurovascular bundles was identified, and no nerve sparing was referenced.

### 2.4. Statistics

*Descriptive statistics:* Continuous variables were analyzed with *t*-tests and Mann–Whitney tests in case of skewed distributions. Categorical variables were analyzed with Fisher’s exact tests.

*Generalized estimation equations* (GEEs) were used to identify independent pre-treatment variables that were significant predictors of the rates of overall problems and the DSSs. The GEE is a multivariate binary logistic (overall problems present/absent) or linear regression (DSSs) model of dependent variables at follow-up, examining associations with independent variables assessed at pre-treatment [19]. Independent variables examined in the GEE were significantly associated with overall problems or DSSs at follow-up: age at pre-treatment, D’Amico risk groups, nerve sparing, neuroticism, not living with partner, co-morbidity, and EPIC-26 overall problem rates and DSSs at pre-treatment. Since age at diagnosis correlated highly with work status, the latter variable was not included in the GEE analyses. The strength of associations in the GEE analyses was expressed by beta coefficients with 95%CIs [19].

The level of significance was set to *p*-values < 0.05, and all tests were two-sided. The data were analyzed with SPSS for PC version 26 (IBM, Armonk, NY, USA).

## 3. Results

### 3.1. Patients

In all, 688 patients had RP, and among them, 675 completed the neuroticism part of the questionnaire at pre-treatment. At three years’ follow-up, 13 patients were deceased, and 551 (83%) delivered new questionnaires. Since biochemical relapse (having two or more PSA-values of >0.2 µg/L after RP) [20] implied additional treatment with radiotherapy and/or hormones, 89 (16%) men who relapsed before follow-up were omitted from the analyses. The sample examined therefore consisted of 462 men.

### 3.2. Rate of High Neuroticism

According to our definition 97 men had high neuroticism (21.0%, 95%, CI 17.3–24.7%), and 365 men had low neuroticism.

### 3.3. Cross-Sectional Comparisons between the Neuroticism Groups

Table 1 (left part) displays the differences between the high and low neuroticism groups at pre-treatment. No significant between-group differences were observed for PCa-related, socio-demographic, or health-related variables, and the same was observed at follow-up (Table 1, right part).

At pre-treatment, the high neuroticism group had significantly higher rates of overall urinary and sexual problems, but not overall bowel problem compared to the low neuroticism group (Table 2, left part). All DSSs were also significantly lower in the high neuroticism group. The high neuroticism patients reported significantly more dysfunction problems on most of the urinary and sexual items and some of the bowel items than men in the low neuroticism group.

At follow-up, patients of the high neuroticism group reported significantly higher rates of overall bowel problems and more problems on most bowel items compared to the low neuroticism group (Table 2, right part). Overall urinary and sexual problems did not show significant between-group differences with few significant differences in the problems on the single items scores of these domains. The irritation/obstruction and bowel DSSs were significantly lower at follow-up in the high neuroticism group, while no significant differences were observed concerning the DSSS of urinary leakage or sexual domain.

### 3.4. Predictors for Overall Problem Rates and Mean Scores at Follow-Up

For both overall urinary, bowel, and sexual problems at follow-up, corresponding problems at pre-treatment were the strongest significant positive predictors (Table 3). In addition, at follow-up, high neuroticism predicted more bowel problems and bilateral nerve sparing less sexual problems with no nerve sparing as reference.

Concerning all DSSs at follow-up, the corresponding DSSs at pre-treatment again were the strongest significant positive predictors (Table 4). High neuroticism was a significant predictor of lower urinary irritation/obstruction and bowel DSSs at follow-up. Younger age was a significant predictor of lower urinary leakage DSSs/High D’Amico risk group and bilateral nerve sparing were significant predictors of better sexual DSSs compared to their references at follow-up. Younger age at diagnosis was a significant predictor of less severe urinary leakage and sexual DSSs.

## 4. Discussion

In our sample, 21% of the men who had RP for PCa reported high neuroticism at pre-treatment. Related to our research questions we first observed that men with high neuroticism at pre-treatment reported significantly higher rates of overall urinary and sexual problems, while the difference for bowel problems was close to significant (*p* = 0.06). Significant between-group differences were found for most urinary and sexual items, and for some bowel items. All pre-treatment DSSs were significantly lower (worse) in the high neuroticism group.

At follow-up, those with high neuroticism had significantly more overall bowel problems, while the differences were non-significant for overall urinary and sexual problems and their corresponding items. The high neuroticism group also had lower bowel and urinary irritation/obstruction DSSs. For all overall problems and all DSSs at follow-up, the corresponding measures at pre-treatment were significant positive predictors. High neuroticism at pre-treatment predicted more overall bowel problems and worse bowel and irritation/obstruction DSSs at follow-up.

The predictive power of pre-treatment sexual function, age, and nerve sparing for erectile function two years after RP has been demonstrated previously [21]. Pre-treatment overall sexual problems significantly predicted the same measure after more than a year of follow-up in men who had RP [22]. As to the number of patients with overall urinary and bowel problems and urinary- and bowel-related DSSs after RP, we found no predictive studies of the corresponding pre-treatment measurements. However, in combined samples of patients treated with RP or radiotherapy, pre-treatment overall problems and DSSs were predictive of these variables two years later [13,23]. We confirmed that older age at diagnosis was a significant predictor of worse urinary leakage and worse sexuality after RP [24].

Compared to our previous prospective study of neuroticism in men treated with RP and radiotherapy at 24 months’ follow-up for localized PCA [13], the present study supplements at three years’ follow-up that overall problems and DSSs at pre-treatment were significant predictors of these variables at three years’ follow-up. In another previous paper presenting findings from the NUCG VII study, our group reported that “any neuroticism” at pre-treatment was a significant predictor of overall sexual problems (bother) at one-year follow-up [12], and a similar prediction was not observed by the current study at three years’ follow-up. However, that study used a cut-off ≥1 for “any neuroticism”, giving a prevalence of 41%, while we used a cut-off ≥2 with a prevalence of 21% for “high neuroticism”. Use of different statistical methods for predictor analyses in that paper and the present study could also explain why the findings differ.

An interesting finding is that overall sexual problems and most functional sexual issues are significantly more common at pre-treatment in the high neuroticism versus the low neuroticism group. However, at follow-up, these group differences are non-significant. Our tentative explanation is that sexual problems and functions at pre-treatment are mostly determined by psychological factors, i.e., neuroticism and corresponding anxiety and depression [10]. After RP, anatomical factors become much stronger, and therefore, the between-group differences become non-significant at follow-up. We presume that the same explanation also can be applied to the findings concerning urinary problems and functions.

In contrast, the rate of overall bowel problems remained associated with high neuroticism, which then can be viewed as a significant predictor of such problems. The explanation could be that the bowel system hardly is affected by the anatomical changes of RP leaving more influence on high neuroticism. Another explanation could be the fact that bowel function is strongly influenced by mental factors through the so-called “bladder–gut–brain” axis [25]. However, the proportions of men with overall bowel problems are <8% of the sample both at pre-treatment and follow-up, casting doubt on the validity of this finding.

Other studies of neuroticism in PCa patients have cross-sectional designs. Perry et al. [26] demonstrated that emotional distress, depression, and suicidal ideation were significantly associated with high neuroticism in a heterogeneous PCa sample. Gerhart et al. [27] found that men with PCa and high neuroticism showed more depression, anxiety, and worry compared to men with low neuroticism. However, none of these studies included analyses of overall problems (bother). As to pre-treatment findings on the EPIC-26 in men with PCa, a recent German study only reported on dysfunctional AOHs and not on overall problems [28].

Personality traits such as neuroticism, are rated as “how you *usually* behave, feel, or act” in contrast to transient states represented by anxiety and depression based on scores of the last 1–2 weeks. Personality traits are stable cognitive and emotional reaction patterns finally set during childhood and adolescence but somewhat modified later in life [4]. Recently, neuroticism and other basic personality traits are considered as more modifiable through psychological and pharmacological interventions and systematic training [5]. These findings give more optimism concerning modifications of high neuroticism and warrant referral of motivated patients to such interventions.

Since high neuroticism is significantly associated with overall problems and DSSs both at pre-treatment and follow-up, clinicians responsible for men after RP should be attentive to high neuroticism as a risk factor for increased problem experience. Eventually, urologists should consider if completion of a short screening PROM for neuroticism (see Supplementary Materials) should be part of the pre-operative evaluation procedure as a supplement to the EPIC-26. However, our findings also support the statement that having any overall problems or low DSSs at pre-treatment implies an increased risk for similar problems and DSSs three years later. This fact and identification of high neuroticism should be themes during the pre-treatment counseling of men with PCa.

Our study had a considerable sample size, and we also considered the prospective design, use of established PROMs with documented psychometric properties, and use of the GEE statistics as strengths of our study.

A limitation of our study was that the participants only rated neuroticism at pre-treatment and not at follow-up. Another limitation was that the psychological data were based on questionnaire responses rather than on psychological evaluation performed by interviews.

In conclusion, the current study only weakly supported high neuroticism as a predictor of overall bowel problems and bowel DSS at three years’ follow-up. However, high neuroticism was significantly associated with higher overall problem rates and lower DSSs both at pre-treatment and follow-up. Screening for neuroticism could therefore be helpful for the clinicians in their pre-treatment counseling of PCa patients regarding overall problems and low DSSs after RP.

## Figures and Tables

**Table 1:** Characteristics of the high and low neuroticism groups at pre-treatment and follow-up (N = 462).

Variables	Pre-Treatment			Follow-Up		
	High Neuroticism N = 97	Low Neuroticism N = 365	*p*	High Neuroticism N = 97	Low Neuroticism N = 365	*p*
Age at diagnosis, mean (SD)	62.3 (5.2)	62.9 (5.4)	0.29			
Age at survey, mean (SD)				65.4 (5.1)	66.0 (5.4)	0.30
Follow-up time, mean (SD)				3.1 (0.3)	3.1 (0.4)	0.87
D’Amico categories, N (%)			0.46			
*Low risk*	15 (15)	73 (20)				
*Intermediate risk*	59 (61)	198 (54)				
*High risk*	23 (24)	94 (26)				
Nerve sparing			0.10			
*None*	36 (37)	166 (46)				
*Unilateral*	34 (35)	89 (24)				
*Bilateral*	27 (28)	108 (30)				
>12 years’ education, N (%)	51 (53)	196 (54)	0.25			
Living with partner, N (%)	82 (85)	320 (91)	0.08			
*Work status, N (%)*			0.75			
*Paid work*	59 (62)	213 (60)				
*Pensioned*	37 (38)	144 (40)				
Co-morbidity, N (%)			0.48			0.56
*None*	59 (62)	245 (68)		79 (81)	300 (82)	
*1 disease*	30 (32)	92 (25)		14 (14)	57 (16)	
*≥2 diseases*	6 (6)	25 (7)		4 (5)	8 (2)	

**Table 2:** EPIC-26 rates of problems and DSSs of the high and low neuroticism groups at pre-treatment and follow-up (N = 462).

Variables	Pre-Treatment			Follow-Up		
EPIC-26 Problems, N (%)	High Neuroticism N = 97	Low Neuroticism N = 365	*p*	High Neuroticism N = 97	Low Neuroticism N = 365	*p*
Urinary domain						
Leakage (Q1)	15 (16)	18 (5)	<0.001	45 (47)	138 (39)	0.134
Lack of control (Q2)	1 (1)	5 (1)	0.791	11 (12)	29 (8)	0.300
Pad use (Q3)	3 (3)	0 (0)	0.009	17 (18)	53 (15)	0.479
Dripping (Q4a)	11 (12)	15 (4)	0.008	34 (36)	101 (29)	0.166
Pain (Q4b)	8 (8)	9 (3)	0.009	9 (10)	10 (3)	0.004
Bleeding (Q4c)	1 (1)	3 (1)	0.860	6 (6)	12 (3)	0.195
Weak stream (Q4d)	41 (44)	104 (30)	0.012	22 (23)	54 (15)	0.066
Frequent need (Q4e)	51 (53)	135 (38)	0.009	44 (46)	115 (32)	0.012
*Urinary problem (Q5)*	38 (40)	88 (25)	0.004	32 (34)	103 (29)	0.378
Bowel domain						
Urgency (Q6a)	7 (7)	11 (3)	0.061	8 (8)	7 (2)	0.002
Increased frequency (Q6b)	13 (14)	17 (5)	0.002	7 (7)	13 (4)	0.129
Loss of control (Q6c)	3 (3)	4 (1)	0.161	7 (7)	4 (1)	0.001
Bloody stools (Q6d)	1 (1)	5 (1)	0.779	4 (4)	3 (1)	0.020
Pain (Q6e)	15 (16)	22 (6)	0.003	16 (17)	13 (4)	<0.001
*Bowel problem (Q7)*	12 (12)	24 (7)	0.062	13 (14)	22 (6)	0.015
Sexual domain						
Erectile problem (Q8a)	59 (63)	178 (49)	0.018	80 (85)	303 (89)	0.359
Orgasmic problem (Q8b)	57 (61)	149 (41)	0.001	72 (77)	248 (73)	0.428
Poor quality erections (Q9)	18 (19)	42 (12)	0.068	61 (65)	190 (56)	0.105
Infrequent erections (Q10)	37 (39)	83 (23)	0.002	72 (77)	244 (71)	0.313
Poor sexual function (Q11)	70 (75)	227 (62)	0.028	87 (93)	304 (89)	0.301
*Sexual problem (Q12)*	48 (51)	130 (36)	0.008	66 (70)	248 (70)	0.889
EPIC-26 DSSs (SD)						
*Urinary leakage*	82.0 (16.4)	86.1 (9.8)	<0.001	68.8 (26.8)	73.5 (27.0)	0.067
*Urinary irritation/obstruct*	77.6 (17.1)	84.0 (15.3)	<0.001	82.8 (16.1)	89.2 (13.8)	<0.001
*Bowel domain*	92.9 (12.1)	96.0 (8.1)	0.018	92.3 (14.3)	96.8 (7.1)	0.003
*Sexual domain*	57.9 (27.4)	69.7 (29.7)	0.001	28.0 (30.2)	32.3 (29.3)	0.214

**Table 3:** General estimating equations (GEEs) estimates of predictors of overall problem present at 3-year follow-up.

Variables	Overall Urinary Problem Present B 95%CI Wald p	Overall Bowel Problem Present B 95%CI Wald p	Overall Sexual Problem Present B 95%CI Wald p
*D’Amico risk groups*			
Low (reference)	0.0	0.0	0.0
Intermediate	0.17 −0.42–0.75 0.58	−0.43 −1.38–0.51 0.37	0.20 −0.34–0.73 0.47
High	0.43 −0.24–1.09 0.21	−1.03 −2.20–0.15 0.09	−0.10 −0.73–0.53 0.76
*Nerve sparing*			
None (reference)	0.0	0.0	0.0
Unilateral	−0.40 −0.94–0.15 0.15	0.16 −0.72–1.04 0.72	−0.17 −0.68–0.35 0.53
Bilateral	0.32 −0.19–0.83 0.21	−0.14 −1.08–0.80 0.77	−0.68 −1.20–−0.16 0.01
Non-paired relation	0.31 −0.38–0.99 0.38	0.44 −0.76–1.64 0.47	−0.34 −1.00–0.32 0.32
Short education	−0.04 −0.49–0.42 0.87	−0.60 −1.43–0.24 0.16	−0.13 −0.57–0.30 0.55
Age at diagnosis	0.01 −0.03–0.05 0.60	0.05 −0.03–0.12 0.19	0.01 −0.04–0.05 0.77
High neuroticism	0.12 −0.40–0.64 0.65	0.87 0.05–1.69 0.04	−0.14 −0.68–0.40 0.61
*Co-morbidity*			
None (reference)	0.0	0.0	0.0
1 disease	−0.39 −0.90–0.13 0.14	−0.14 −0.98–0.70 0.74	0.44 −0.07–0.94 0.09
≥2 diseases	−0.50 −1.36–0.37 0.26	−0.11 −1.17–0.95 0.84	−0.04 −0.92–2.81 0.93
Problem at pre-treatment	0.64 0.16–1.11 0.009	2.63 1.64–3.62 <0.001	1.01 0.52–1.50 <0.001

**Table 4:** General estimating equations (GEEs) estimates of predictors of domain summary scores (DSSs) at 3-year follow-up.

Variables	Urinary Leakage Symptom Domain Score B 95%CI Wald p	Urinary Irritation/Obstruction Symptom Domain Score B 95%CI Wald p	Bowel Symptom Domain Score B 95%CI Wald p	Sexual Symptom Domain Score B 95%CI Wald p
*D’Amico risk groups*				
Low (reference)	0.0	0.0	0.0	0.0
Intermediate	−1.81 −8.58–4.97 0.60	−0.70 −3.88–2.50 0.67	1.49 −0.51–3.50 0.15	−2.58 −11.47–2.16 0.18
High	−1.25 −9.16–6.66 0.76	–1.11 −4.60–2.38 0.53	1.75 −0.42–3.93 0.11	−9.13 −16.91–1.36 0.02
*Nerve sparing*				
None	0.0	0.0	0.0	0.0
Unilateral	0.86 −5.13–6.85 0.78	−1.08 −4.14–1.97 0.49	1.09 −0.86–3.30 0.28	−5.63 −11.60–0.33 0.06
Bilateral	−4.55 −11.04–1.94 0.17	−1.39 −4.73–1.96 0.42	0.87 −0.79–2.52 0.30	−6.64 −12.52–−0.77 0.03
Non-paired relation	−6.05 −15.14–3.04 0.19	−5.32 −9.87–−0.76 0.022	−1.94 −5.76–1.88 0.32	−2.23 −10.36–5.89 0.59
Short education	0.53 −4.69–5.74 0.84	−2.23 −4.89–0.44 0.10	1.15 −0.41–2.70 0.15	−1.44 −6.50–3.62 0.58
Age at diagnosis	−0.63 −1.12–−0.15 0.01	−0.01 −0.24–0.2 0.90	−0.04 −0.16–0.08 0.56	−0.95 −1.46–−0.44 <0.001
High neuroticism	−2.61 −8.37–3.14 0.37	−4.16 −7.74–0.58 0.023	−2.88 −5.07–−0.69 0.01	1.37 −4.95–7.70 0.67
*Co-morbidity*				
None (reference)	0.0	0.0	0.0	0.0
1 disease	3.69 −2.26–9.64 0.22	2.33 −0.75–5.41 0.14	−0.98 −2.71–0.75 0.27	−2.99 −11.50–5.52 0.49
≥2 diseases	6.00 −1.86–13.86 0.14	−2.87 −7.70 −1.95 0.24	−3.20 −6.53–0.13 0.06	−0.41 −8.46–7.64 0.92
DSS at pre-treatment	0.39 0.12–0.66 0.005	0.23 0.12–0.34 <0.001	0.50 0.35–0.66 <0.001	0.45 0.36–0.55 <0.001

## Data Availability

According to Norwegian data legislation, the data of this study cannot be made generally available. Requests should be sent to the corresponding author.

## Supplementary Materials

The following supporting information can be downloaded at: https://www.mdpi.com/article/10.3390/curroncol29080459/s1, See Supplementary Materials File.
