# Supplementary material for: High Neuroticism Is Related to More Overall Functional Problems and Lower Function Scores in Men Who Had Surgery for Non-Relapsing Prostate Cancer
Source: Curr Oncol. 2022 Aug 17;29(8):5823–32. doi: 10.3390/curroncol29080459 (PMC9406934; doi:10.3390/curroncol29080459)
Supplement: Supplementary file 1 [file curroncol-29-00459-s001.zip › curroncol-1851583-supplementary.pdf]

Supplement.

The 6-item version of neuroticism based on the Eysenck Personality Questionnaire.

Instruction: The items below concern how you usually behave, feel, or act. Please, set a ring round the number for either Yes or No for each item. Please, respond quickly and do not think too long about the meaning of each item.

Items	Yes	No
1. Are you often worried?	1	0
2. Are your feelings easily hurt?	1	0
3. Do you often feel that you lose interest?	1	0
4. Do you have nervous problems?	1	0
5. Do you often feel tired and indifferent/unmotivated without reason?	1	0
6. Do you worry that terrible things might happen?	1	0

Low neuroticism score: sum score 0 – 2; High neuroticism score: sum score 3 – 6) (reference #14).
